# Fabrication of light trapping structures specialized for near-infrared light by nanoimprinting for the application to thin crystalline silicon solar cells

**DOI:** 10.1186/s11671-023-03840-6

**Published:** 2023-05-03

**Authors:** Yuto Kimata, Kazuhiro Gotoh, Satoru Miyamoto, Shinya Kato, Yasuyoshi Kurokawa, Noritaka Usami

**Affiliations:** 1 Department of Materials Process Engineering, Graduate School of Engineering, Nagoya University, Furo-cho, Chikusa-ku, Nagoya, 464-8603 Japan; 2 Department of Electrical and Mechanical Engineering, Nagoya Institute of Technology, Showa-ku, Nagoya, 466-8555 Japan; 3 Institutes of Innovation for Future Society, Nagoya University, Furo-cho, Chikusa-ku, Nagoya, 464-8601 Japan

**Keywords:** Solar cell, Light trapping, Nanoimprint, Colloidal lithography, Dry etching

## Abstract

Vehicle-integrated photovoltaics (VIPV) are gaining attention to realize a decarbonized society in the future, and the specifications for solar cells used in VIPV are predicated on a low cost, high efficiency, and the ability to be applied to curved surfaces. One way to meet these requirements is to make the silicon substrate thinner. However, thinner substrates result in lower near-infrared light absorption and lower efficiency. To increase light absorption, light trapping structures (LTSs) can be implemented. However, conventional alkali etched pyramid textures are not specialized for near-infrared light and are insufficient to improve near-infrared light absorption. Therefore, in this study, as an alternative to alkaline etching, we employed a nanoimprinting method that can easily fabricate submicron-sized LTSs on solar cells over a large area. In addition, as a master mold fabrication method with submicron-sized patterns, silica colloidal lithography was adopted. As a result, by controlling silica coverage, diameter of silica particles (*D*), and etching time (*t*_et_), the density, height, and size of LTSs could be controlled. At the silica coverage of 40%, *D* = 800 nm, and* t*_et_ = 5 min, the reduction of reflectance below 65% at 1100 nm and the theoretical short-circuit current gain of 1.55 mA/cm^2^ was achieved.

**Supplementary Information:**

The online version contains supplementary material available at 10.1186/s11671-023-03840-6.

## Introduction

In recent years, VIPVs have attracted much attention to achieve carbon neutrality [1–4]. One approach to achieve long distances to drive is an implementation of highly efficient tandem solar cells on VIPVs. Schygulla et al. [5] reported a two-terminal wafer-bonded III–V//crystalline Si (c-Si) triple-junction solar cell with the efficiency of 35.9%. Recently, a 32.5% efficient 2-terminal (2T) Si/perovskite tandem solar cell has been reported [6]. To implement solar cells on curved surfaces like a roof and hood of vehicles, solar cells should be flexible and a silicon substrate must be thin. Sai et al. fabricated a flexible Si heterojunction solar cell with a thickness of approximately 50 μm [7]. However, the challenge is that thin silicon substrates do not absorb near-infrared light well enough. In general, alkaline etching [8] and photolithography [9, 10] have been used to fabricate optical confinement structures. However, the alkaline etching method is insufficient to absorb near-infrared light, and it is difficult to fabricate on thin silicon substrates and in a large area. Photolithography also has the problem of low throughput. There are some other approaches to enhance the near-infrared light absorption [11–14]. Aluminum anodization process is one of them [15–18]. High density honeycombed nano-pattern with sub-wavelength size can be obtained from anodic aluminum oxide (AAO) and it can be used for anti-reflection structure. Another approach is the nanoimprinting technique, which enables us to control the morphology of submicron-sized light trapping structures (LTSs) and to fabricate the LTSs on a thin Si substrate in a large area [19–27]. Various designs of LTSs have been fabricated by nanoimprinting, including a binary diffraction grating [23] and inverted pyramid shapes [24]. Although conventional LTSs fabricated by nanoimprinting technique are periodic, non-periodic LTSs will enhance light absorption in a wide wavelength range. For example, conventional random pyramid texture for c-Si solar cells [8] and ZnO texture structure for Si-based or chalcopyrite thin film solar cells [28, 29] are reported.

In this study, to obtain non-periodic LTSs, the fabrication of master molds by colloidal lithography using silica particles [23] was combined with nanoimprinting. The colloidal lithography has the advantage of easily controlling the morphology of LTSs in a submicron range and cost reduction and large-area fabrication. The diameter and height of LTSs were controlled by varying the diameter of silica particles (*D*) and etching time (*t*_et_). The silica coverage was controlled by varying the concentration and deposition time of silica solution and spin-coating speed. Non-periodic LTSs were obtained by non-periodic adsorption of silica particles on a Si substrate and dry etching. The combination of colloidal lithography and nanoimprinting for solar cell applications has not been reported yet. We implemented the LTSs on the backside of c-Si/ITO substrates by nanoimprinting technique and evaluated the light confinement performance.

## Experimental methods

**Fig. 1:**
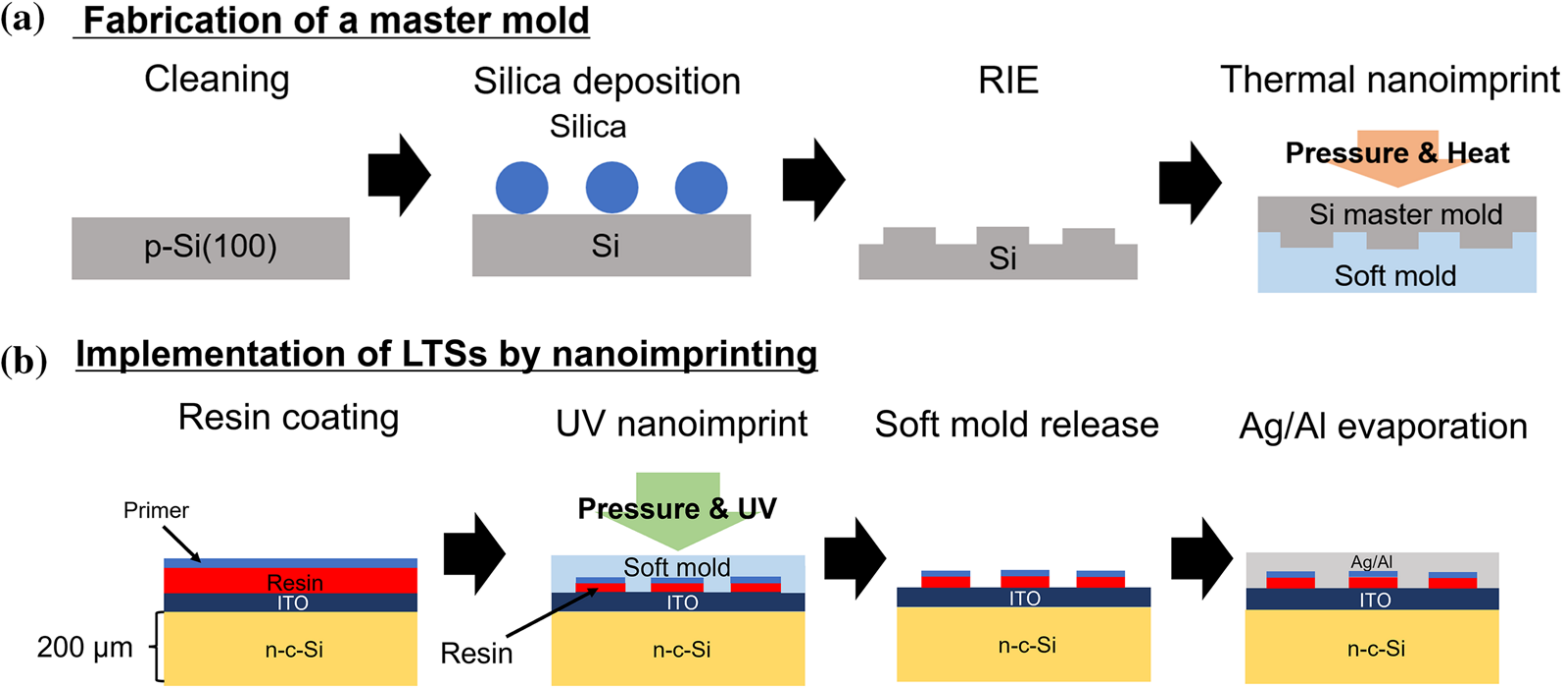
Schematic diagram of **a** the fabrication process of a master mold for LTSs and **b** implementation process of LTSs on a back side of solar cells by nanoimprinting

The fabrication process of a master mold of LTSs is shown in Fig. 1a. A 2 × 2 cm^2^ c-Si substrate was used. c-Si substrates were cleaned by the RCA process. Subsequently, they were immersed in 5% hydrofluoric acid for 1 min to remove a natural oxide. The substrates were immersed in ethylenediamine and irradiated with UV light for 3 h. After that, ethylenediamine was removed with toluene, ethanol, and deionized (DI) water. Silica particles terminated by carboxyl groups (sicastar^®^, micromod Partikeltechnologie GmbH) were diluted by DI water into 0.1 to 10 wt%. The silica particles with the diameters of 500, 800, 1000, and 1500 nm were used. The colloidal solution of silica particles was stirred with DI water in a homogenizer (Digital Sonifier BRANSON) for 30 min. The deposition of silica particles was carried out by the following two-step method. In our previous work, silica particles terminated by carboxyl groups were sparsely adsorbed on a Si substrate terminated by amino groups, and the density was controlled by controlling the pH in the silica solution [27]. We tried to use the adsorbed silica particles as aggregation nuclei during the second spin-coating. During the second spin-coating, silica particles were attracted to and adsorbed to already adsorbed silica particles. Since the adsorbed silica particles became the aggregation nuclei, the density of silica particles was increased and the silica coverage was also increased. By the two-step method, non-periodically adsorbed silica particles could be obtained. At the first step, 100 µL of this solution was deposited on the c-Si substrate to fully bond the carboxyl groups on the silica particles to the amino groups on the Si substrate for 10 min. At the second step, the solution was spin-coated on the c-Si substrates at 200 rpm for 10 s and dried at 1000–8000 rpm for 30 s. The substrates were etched by reactive ion etching (RIE; Samco RIE-10NR). During the RIE, the silica particles work as an etching mask. The flow rates of CF_4_ and O_2_ were 80 and 8 sccm, respectively. Plasma power was kept constant at 100 W. The etching time (*t*_et_) was varied from 3 to 25 min. The substrate was immersed in 5% hydrofluoric acid for 1 min to remove the remaining silica particles. The structures of fabricated molds were observed by scanning electron microscopy (SEM; JEOL JSM-7001F).

The implementation process of LTSs on the back side of c-Si/ITO substrates is shown in Fig. 1b. The sample size was 1.7 × 1.7 cm^2^. 100 nm-thick indium tin oxide (ITO) layer was deposited onto mirror-polished Si substrates by radio-frequency (RF) magnetron sputtering. A master mold was transferred to a soft mold by thermal nanoimprinting. The pressure, temperature, and duration were 0.6 MPa, 130 °C, and 1 min, respectively. A primer (mr-APS-1, microresist technology GmbH) was applied to the specimens by spin coating to improve the release from the soft mold. The rotational speed was increased at a constant slope for 60 s and was kept at 5000 rpm for 60 s. After natural drying for 5 min, an UV curable resin (mr-NIL 210, microresist technology GmbH) was deposited by spin coating. Rotation speeds at the first and second steps were 500 rpm for 30 s and 3000 rpm for 25 s, respectively. The thickness of the resin was about 100 nm. The soft mold pattern was transferred to the resin by UV nanoimprinting. The pressure and duration were 0.2 MPa and 1 min, respectively. As shown in Fig. S1, under these nanoimprinting conditions, the morphology of the master mold was successfully transferred to the sample. Silver and aluminum electrodes were deposited on the LTSs. For optical evaluation, the light was incident from the front side of the c-Si/ITO/LTSs/Electrode structure. Reflectance was measured by a spectrophotometer (Jasco V-570). The short-circuit current density (*J*_sc_) was calculated from the reflectance spectra and AM1.5G spectra.

## Results and discussion

**Fig. 2:**
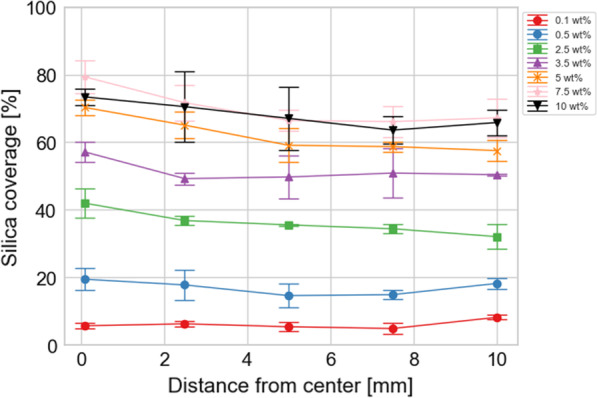
Silica coverage at each point from the center to edge of the substrate. The particle size was 800 nm and the rotation speed was fixed at 8000 rpm. The silica coverage represents the area fraction of the area of adsorbed silica particles to that of Si substrates and was calculated from SEM images as shown in Fig. S2. Five different images were taken near each position on the silicon wafer and the silica coverage was calculated for each image using ImageJ software. Therefore, error bars indicate the minimum and maximum values of the range of five observations

Figure 2 shows the silica coverage at five points on the substrate when the silica concentration was varied from 0.1 to 10 wt%. The particle size was 800 nm and the rotation speed was fixed at 8000 rpm. The silica coverage was estimated using ImageJ software. Binarization was performed to distinguish between silica particles and other regions. The silica coverage is the ratio of the area of all silica particles to a total area of the SEM image. The silica coverage represents the area fraction of the area of adsorbed silica particles to that of Si substrates and calculated from SEM images as shown in Fig. S2. The silica coverage was increased with increasing the silica concentration until 10 wt% at all the positions. The silica coverage started to saturate at the concentration above 5.0 wt%. This is because at a concentration of 5.0 wt% or higher, a monolayer of silica particles was formed almost all over the substrate by spin coating, and excess silica particles could not reach vacancies and were blown away with the solvent. Although the silica coverage at the center of the substrate was slightly higher than that at the other positions, the silica coverage was generally almost uniform, suggesting that the appropriate rotation speed was set for the uniform deposition.

**Fig. 3:**
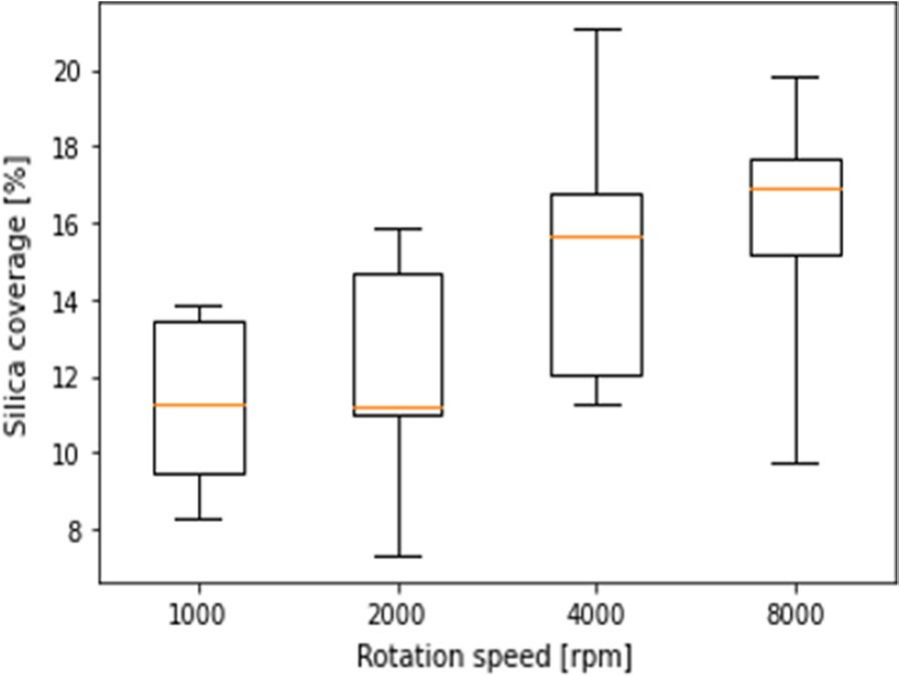
Dependence of silica coverage on rotation speed of spin-coating at the second stage. The particle size was 800 nm and the silica colloid concentration was 0.1 wt%

Figure 3 shows the effect of rotation speed during spin coating on silica coverage. The particle size was 800 nm, and the rotation speed was varied from 1000 to 8000 rpm. The silica solution concentration was 0.1 wt%. The spin coating process is divided into two stages: the first stage is a low-speed process to spread the silica solution uniformly across the substrate, and the second stage is a process to remove the remaining silica solution. Here, the effect of the rotation speed at the second stage on silica coverage was investigated. As the rotation speed was increased, the silica coverage was also increased monotonically. At low rotation speeds, centrifugal force is small and the effect of intermolecular force between silica particles is predominant. Since the migration distance of silica particles is short, if an aggregation nucleus does not exist within the migration distance, the silica particles do not adsorb. Therefore, two kinds of areas where silica particles are aggregated or not are formed. As a result, the silica coverage becomes low. As the rotation speed was increased, the migration distance of silica particles is increased by increasing the centrifugal force. Since the probability for silica particles to adsorb on the Si substrate is increased, the silica coverage is increased. From this result, the rotation speed of 8000 rpm was adopted.

**Fig. 4:**
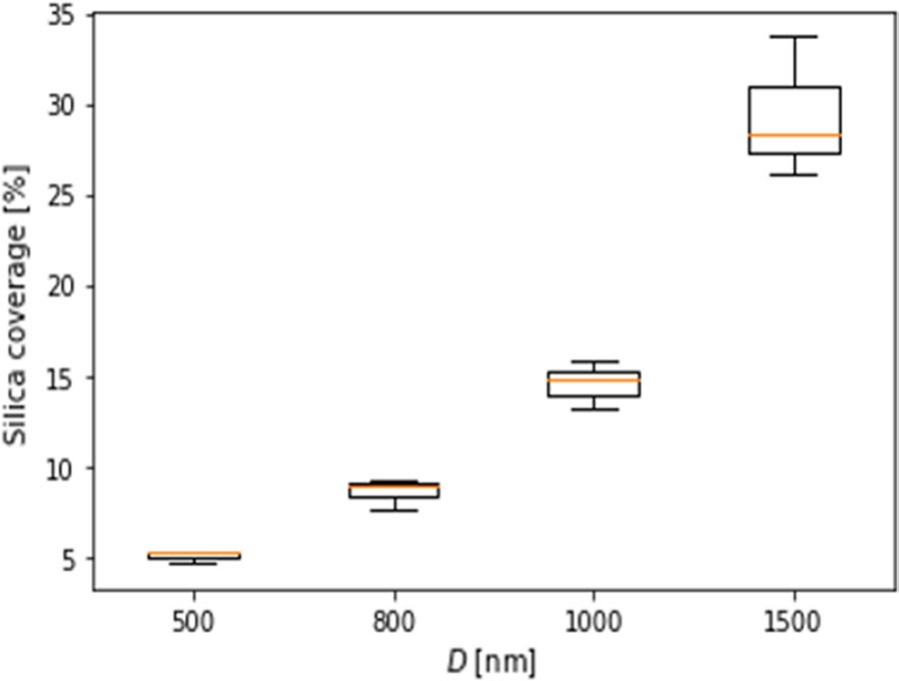
Dependence of silica coverage on *D*. The rotation speed and the silica colloid concentration were 8000 rpm and 0.1 wt%, respectively

**Fig. 5:**
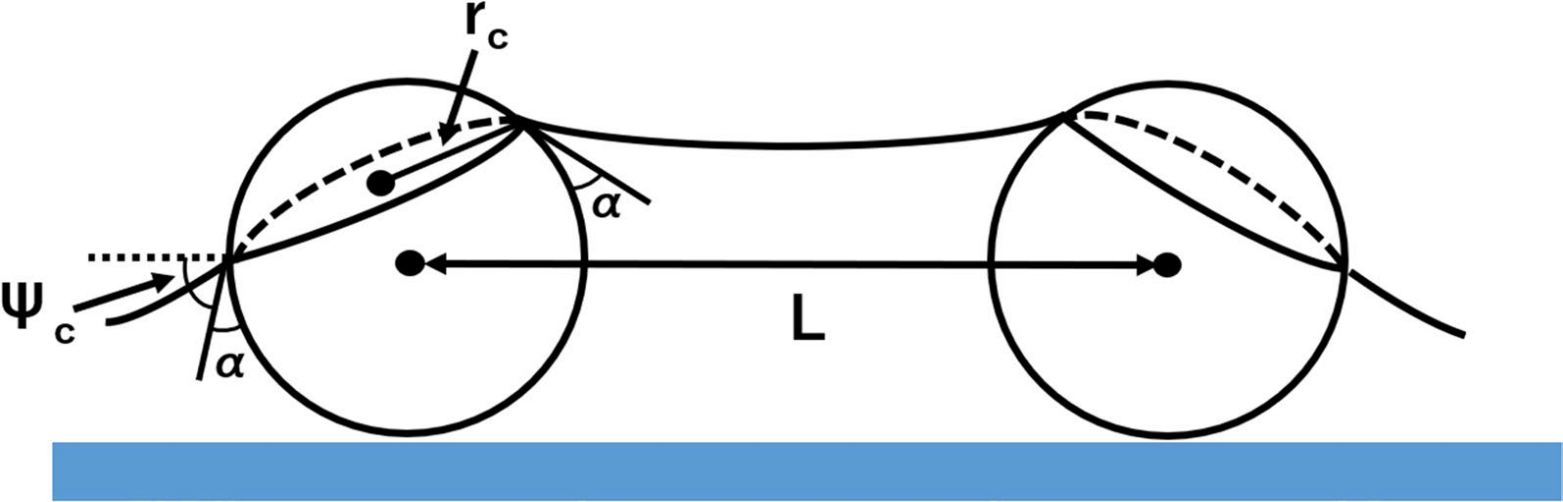
Schematic illustration of two particles partially immersed in a liquid layer for capillary attraction

Figure 4 shows the effect of the size of silica particles on silica coverage. Four silica particle sizes of *D* = 500, 800, 1000, and 1500 nm were used. The rotation speed and the concentration of the silica solution were 8000 rpm and 0.1 wt%, respectively. The silica coverage was increased with increasing silica particle size. Silica particles on a silicon substrate are subject to centrifugal force, convective force, and capillary force (*F*_cap_). Centrifugal force and convective force are related with long-range assembly and *F*_cap_ is related with short-range assembly. Centrifugal force is proportional to the mass of silica particles. Since larger centrifugal force is applied to larger silica particles, the probability to reach capillary assembled region is increased, leading to higher silica coverage. Convective force is caused by hydrodynamic pressure differences due to wetting thickness variation between spots. The spin coating process causes very fast liquid evaporation due to its high-speed spinning. Thus the vapor pressure is a key parameter when selecting a solvent. Since water has a high vapor pressure, rapid liquid evaporation is caused during spin coating. Wetting layer thickness variations are rapidly decreased from center to edge regions on the substrate. Consequently, the effect of the convective force is not so large. Capillary force is the force of attraction between particles and is represented by Eq. (1), which is illustrated in Fig. 5 [30–32].

1 $$F_{cap} = 2\uppi \upgamma {\text{r}}_{c}^{2} (\sin^{2} \Psi _{c} )\frac{1}{L}$$

where,* γ* is the surface tension of the liquid, *r*_c_ is the radius of the three-phase contact line at the particle surface, *Ψ*_c_ is the mean meniscus slope angle at the contact line, and *L* is the interparticle distance. From Eq. (1), capillary force is proportional to *r*_c_^2^. *r*_c_ is increased with increasing *D*. Therefore, *F*_cap_ is increased with increasing *D*.

**Fig. 6:**
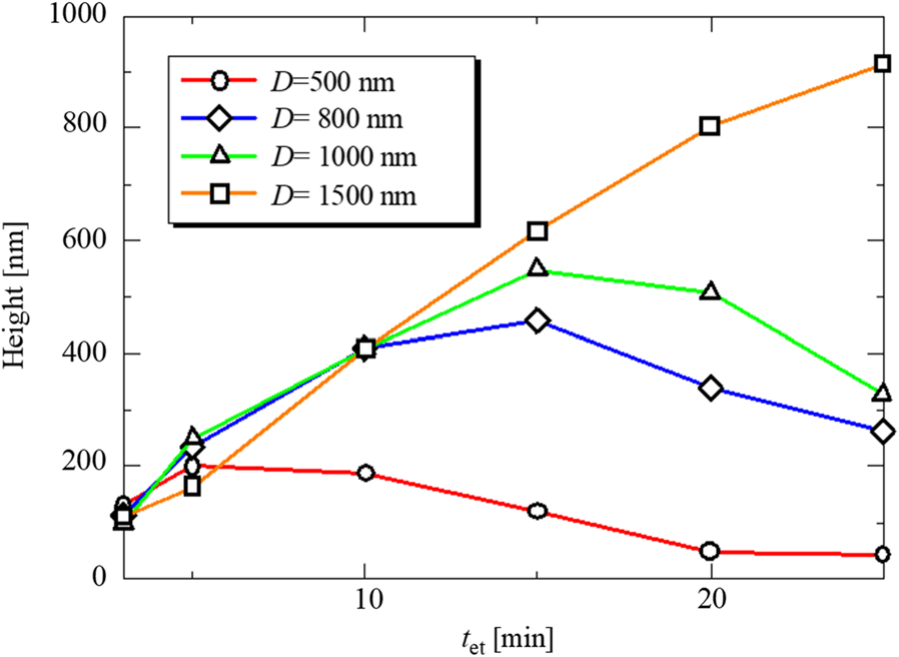
Etching time dependence of the LTS height fabricated by silica particles with a different diameter

**Fig. 7:**
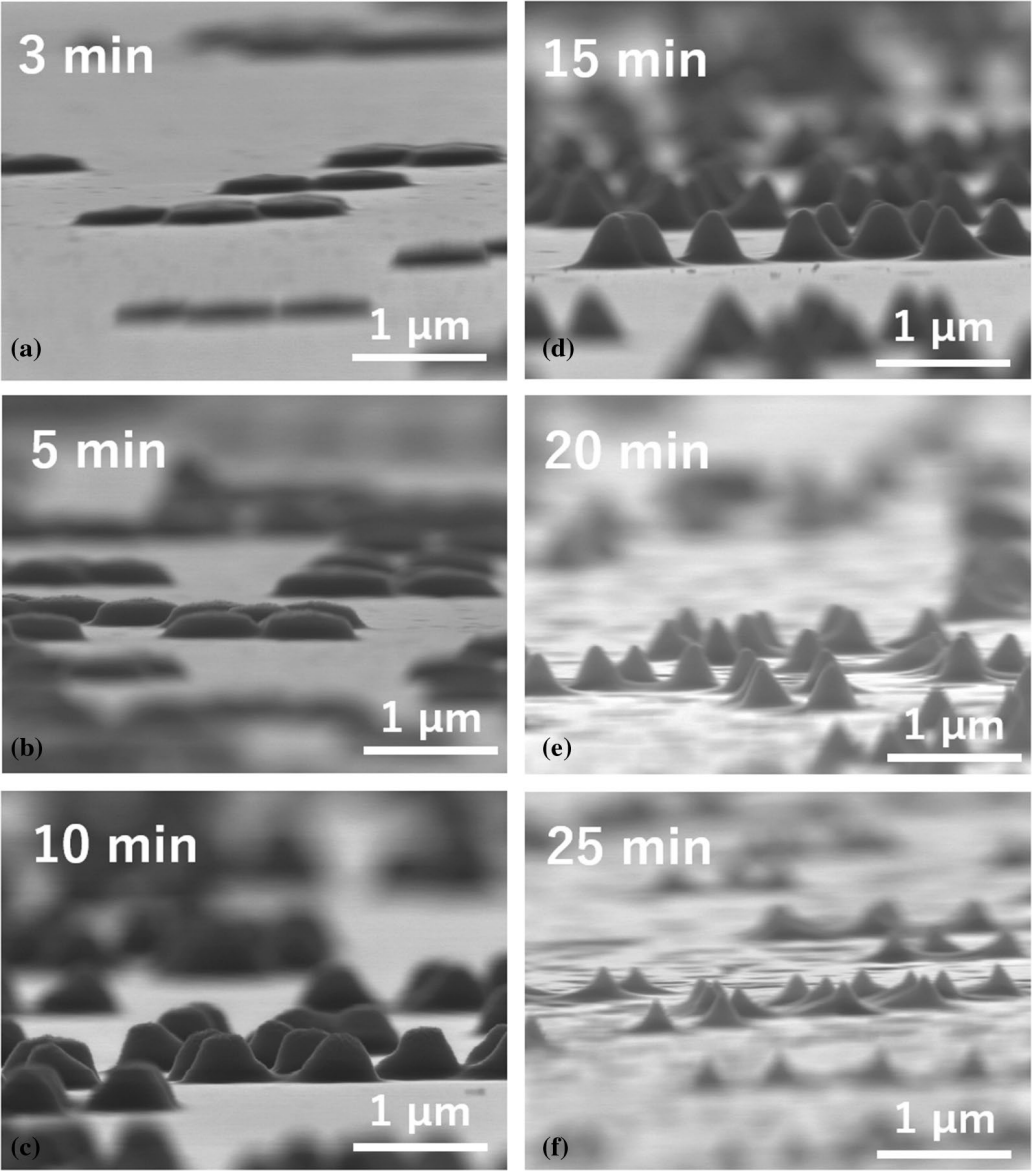
SEM images of LTSs on a master mold fabricated by **a** *D* = 800 nm, *t* = 3 min, **b** *D* = 800 nm, *t* = 5 min, **c** *D* = 800 nm, *t* = 10 min, **d** *D* = 800 nm, *t* = 15 min, **e** *D* = 800 nm, *t* = 20 min, and **f** *D* = 800 nm, *t* = 25 min. Silica concentration was 0.1 wt%

Figure 6 shows the height of LTSs on the Si master molds fabricated by silica particles with different diameters. The rotation speed and the concentration of the silica solution were 8000 rpm and 0.1 wt%, respectively. Regardless of *D*, the height of LTSs has a maximum with respect to *t*_et_. As *D* increases, *t*_et_ when the height of LTSs becomes maximum (*t*_h-max_) increases. Each *t*_h-max_ for *D* = 500, 800, 100, and 1500 nm was 5, 15, 15, and 25 min, respectively. For *D* = 500 nm, the maximum height was about 200 nm at *t*_et_ = 5 min. On the other hand, at *D* = 1000 nm, the maximum height is about 550 nm around *t*_et_ = 15 min. Figure 7 shows SEM images of LTSs at *D* = 800 nm and (a) *t*_et_ = 3, (b) 5, (c) 10, (d) 15, (e) 20 and (f) 25 min. It can be seen that the structure changes from a dome-shape to a cone-shape at 15 min, which corresponds to the *t*_h-max_. Around *t*_et_ = *t*_h-max_, silica particles started to disappear on the LTSs. By the absence of etching masks, the LTSs themselves were etched and the shape was changed into conical shape and the height of the LTSs was decreased. Therefore, *t*_h-max_ is almost same as the time when the mask disappears and depends on the etching selectivity between Si and SiO_2_. Etching selectivity is decided by the flow rate of CF_4_ and O_2_. The higher fraction of oxygen leads to reduce silica etch rates. The etching selectivity was highest at 10% oxygen at 0.63 and decreased slightly with increasing the oxygen fraction [33]. If etching selectivity was decreased, silica particles would disappear at shorter *t* and *t*_h-max_ would be decreased and the height of LTSs would be also decreased.

**Fig. 8:**
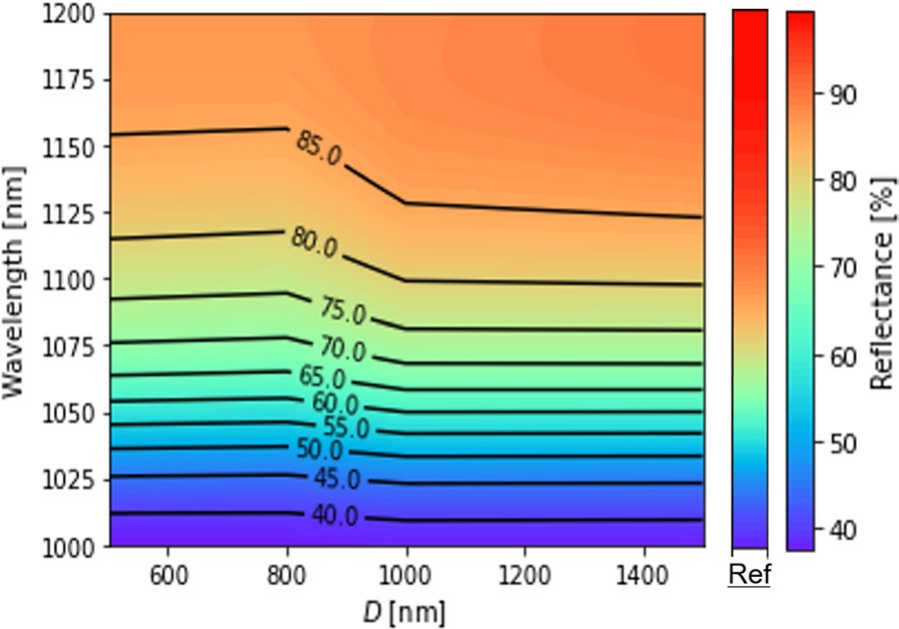
Color mapping of reflectance spectra of LTSs fabricated by silica particles with different *D* at *t*_et_ = 3 min. “Ref” shows the reflectance spectra of the structure of c-Si/ITO/Electrode

Figure 8 shows the reflectance spectra of LTSs fabricated by silica particles with different diameters at *t*_et_ = 3 min. At *t*_et_ = 3 min, the height of LTSs was about 100 nm for all the diameters. In the wavelength range from 1000 to 1200 nm, the reflectance is significantly influenced by the reflection on the back side, since the Ag/Al electrode was deposited on the back side. “Ref” shows the reflectance spectra of the structure of c-Si/ITO/Electrode. The nearly 100% reflectance was measured in infrared light range due to back reflection at the rear electrode and low absorption coefficient of Si. The LTSs reduced the reflectance in all the wavelengths compared to the flat sample. The reflectance at 1200 nm was reduced from 98.9 to 81.3% by the LTS with *D* = 800 nm. The LTSs scattered light and the optical path of the light in the c-Si substrate was increased, leading to the increase in light absorption and the decrease in the reflectance. The reflectance became the lowest at *D* = 500 and 800 nm. This suggests that there is an optimum aspect ratio of LTSs to reduce the reflectance. Sei et al. showed by finite-difference time domain (FDTD) simulation that the incident light is scattered the most effectively by Si LTSs with a diameter of 700 nm [13]. Sai et al. have studied the light-trapping effect of submicron pyramid textures by numerical simulations and showed that an enhanced light-trapping effect occurs because of submicron textures, especially those of similar sizes, as mentioned above [34]. Therefore, the result that *D* = 500 and 800 nm are optimal for LTSs corresponds to the previous results.

**Fig. 9:**
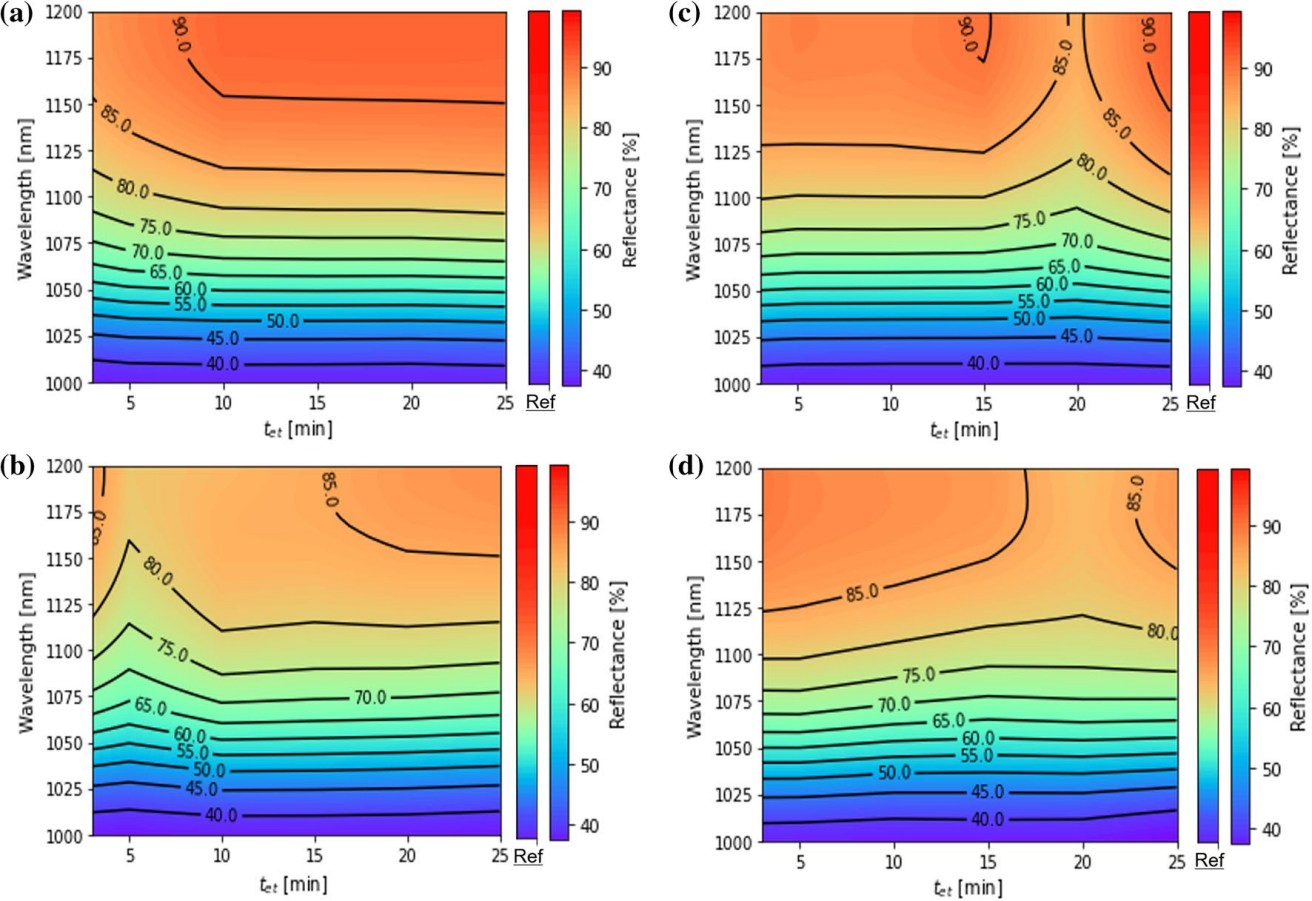
Color mapping of reflectance spectra of LTSs fabricated by silica particles with **a** *D* = 500 nm, **b D** = 800 nm, **c D** = 1000 nm, and **d** *D* = 1500 nm. “Ref” shows the reflectance spectra of the structure of c-Si/ITO/Electrode

Figure 9 shows the reflectance spectra of LTSs fabricated by silica particles with *D* = (a) 500, (b) 800, (c) 1000, and (d) 1500 nm. The *t*_et_ was varied from 3 to 25 min. “Ref” shows the reflectance spectra of the structure of c-Si/ITO/Electrode. At all etching times, the reflectance was lower than that of the Ref sample and the lowest reflectance of 81.4% at 1200 nm was obtained at *D* = 800 nm and *t*_et_ = 5 min. This result corresponds to the previous results. Besides, there is the *t*_et_ when the reflectance was reduced locally in the whole range (*t*_r-min_) for each *D*. Each *t*_r-min_ for *D* = 500, 800, 1000, and 1500 nm was less than 5, 5, 20, and 20 min, respectively. Except for *D* = 1000 nm, *t*_r-min_ is less than *t*_h-max_. One possibility to explain this is the effect of the shape of LTSs. After the height became maximum at *t*_h-max_, the shape of LTSs was changed from a dome-shape into a cone-shape. This indicates that the dome-shaped LTSs may be preferable for light scattering compared to cone-shaped LTSs. Another possibility is the effect of silica coverage. The silica coverage was increased with increasing *D* as shown in Fig. 4. The results in Fig. 9 can be divided into two group; low silica coverage group (G1) and high silica coverage group (G2). *D* = 500 and 800 nm belong to G1 and *D* = 1000 and 1500 nm belong to G2. In the case of G1, the silica coverage is low. The scattering effect of a single dome-shaped LTS becomes important. In the case of G2, the silica coverage is high. In this case, the cone-shaped LTS assembly has high scattering effect which is similar to a random pyramid texture. In this way, there is a possibility that light scattering is complicatedly determined by a combination of not only height but also shape and coverage of LTSs.

**Fig. 10:**
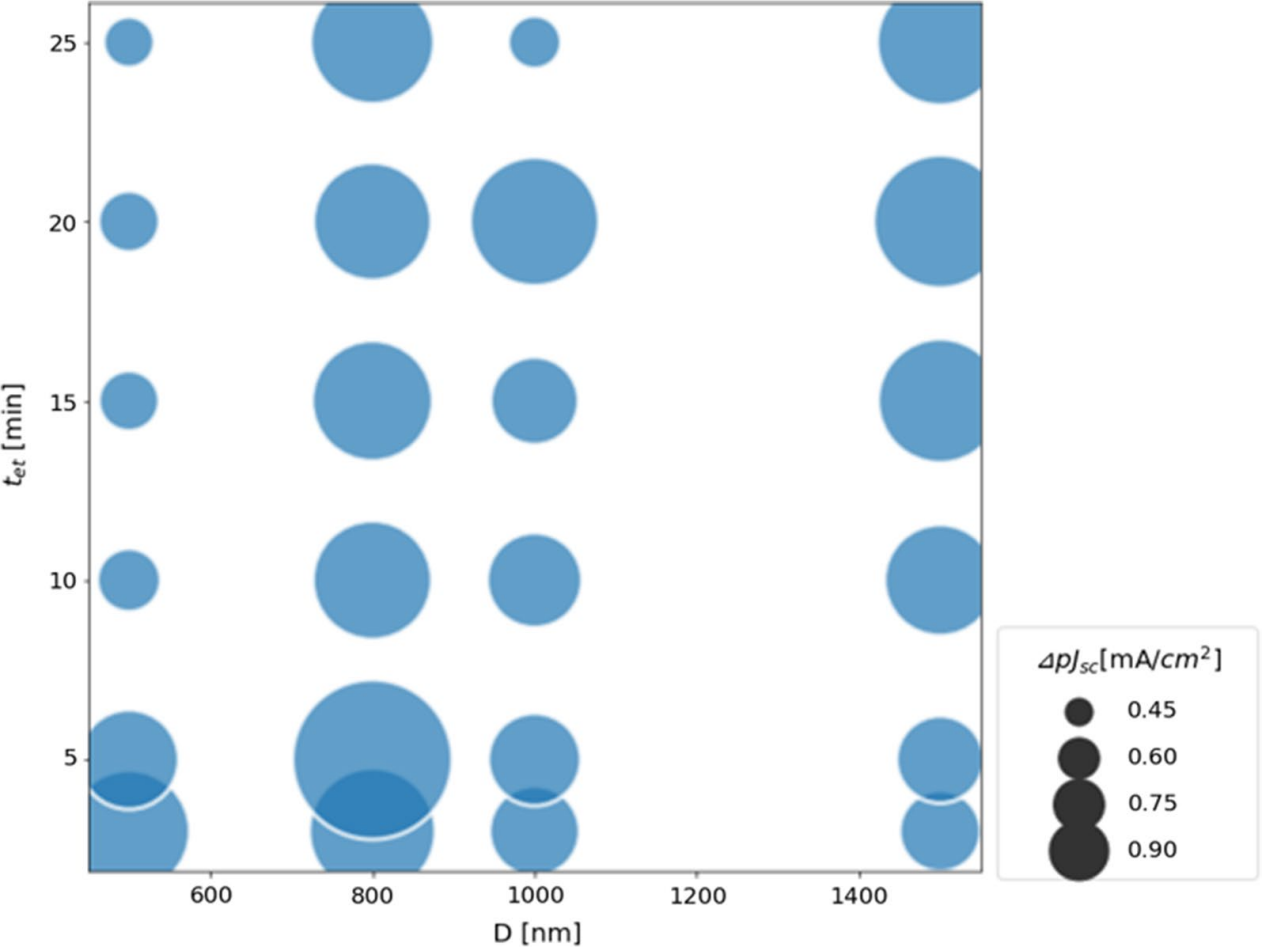
*Δ*p*J*_sc_ calculated from the reflectance spectra shown in Fig. 9 and Eq. (1) as a function of *t*_et_ and *D*. It was assumed that all of the absorbed photons were converted into electron–hole pairs and extracted externally. The area of each circle represents the absolute value of *Δ*p*J*_sc_

The projected *J*_sc_ (p*J*_sc_) was calculated by the reflectance spectra shown in Fig. 8. The p*J*_sc_ was calculated by the following equation,

2 $$pJ_{sc} = \int_{{1000{\text{nm}} }}^{{1200{\text{nm}} }} {qAN_{photon} d\lambda }$$

where *q* is the elementary charge, *λ* is the wavelength of incident light, *A* is the absorptance, and *N*_photon_ is the number of photons in AM1.5G spectrum. It was assumed that all of the absorbed photons were converted into electron–hole pairs and extracted externally. Figure 10 shows the increment of p*J*_sc_ against the Ref sample (*Δ*p*J*_sc_). The p*J*_sc_ was improved compared to the Ref sample at all *D* and *t*_et_. When *t*_et_ was short, *Δ*p*J*_sc_ was higher at smaller diameter. On the other hand, when *t*_et_ was long, *Δ*p*J*_sc_ was higher at larger diameter. This suggests *Δ*p*J*_sc_ was increased when the height of LTSs was lower regardless of the diameter. A maximum gain of 1.00 mA/cm^2^ was observed at *D* = 800 nm and* t*_et_ = 5 min, suggesting that *D* = 800 nm may be the optimal width for near-infrared light in the 1000 to 1200 nm range and lower aspect ratio is preferable.

**Fig. 11:**
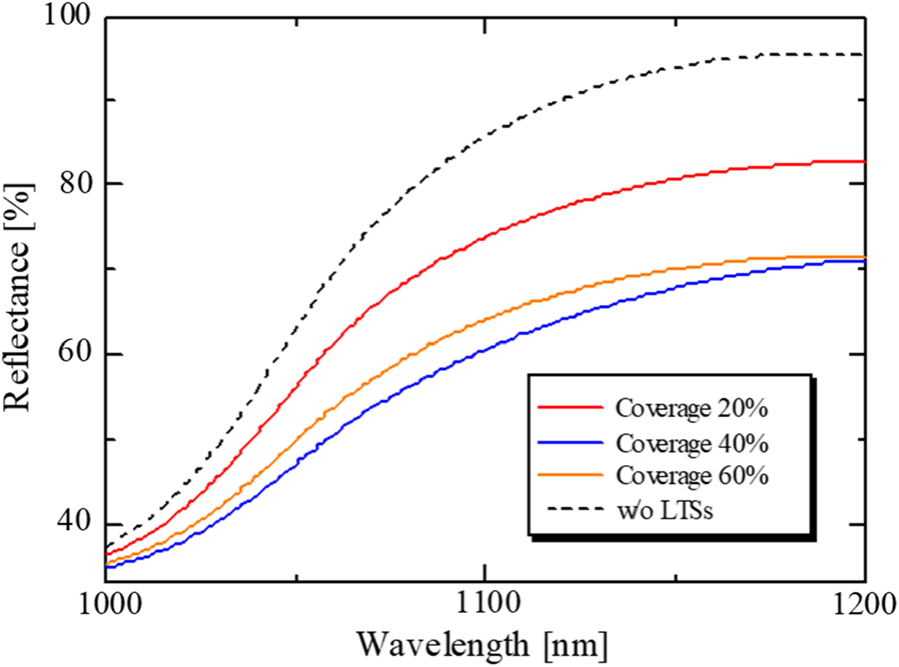
**a** Reflectance spectra of LTSs fabricated by different silica coverages. *D* = 800 nm, rotation speed = 8000 rpm, and* t*_et_ = 5 min were adopted. Silica coverages of 15, 46, and 57% were obtained by the silica concentrations of 0.5, 2.5, and 10 wt%, respectively. The reflectance of the sample with the LTSs in the visible light region was almost same as that without the LTSs as shown in Fig. S4. **b** *Δ*p*J*_sc_ calculated from the reflectance spectra shown in this figure and Eq. (1). It was assumed that all of the absorbed photons were converted into electron–hole pairs and extracted externally. The p*J*_sc_ of the reference cell was 2.34 mA/cm^2^

**Fig. 12:**
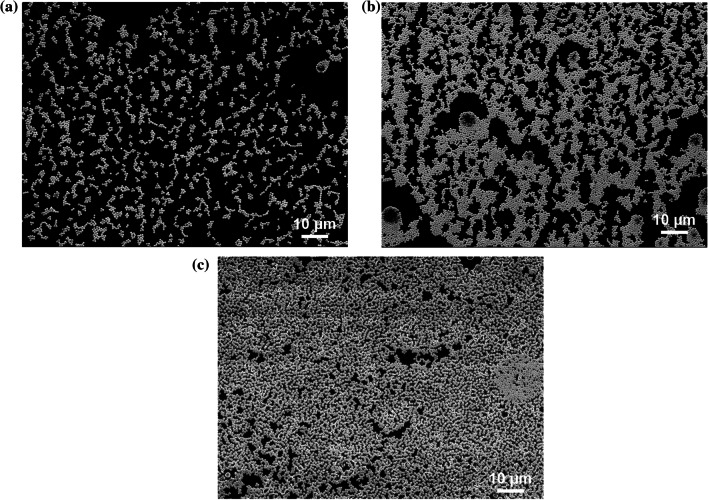
SEM images of master molds prepared with the silica coverage of **a** 16, **b** 45, and **c** 57%

Finally, the effect of silica coverage on the reflectance was investigated. Figure 11 shows reflectance spectra for each silica coverage. *D* = 800 nm, rotation speed = 8000 rpm, and *t*_et_ = 5 min were adopted. Three molds with different silica coverages were prepared by the silica concentrations of 0.5, 2.5 and 10 wt%. The silica coverages of them were 16, 45, and 57%. The mold patterns were transferred to the ITO/Si substrates and reflectance was measured. As shown in Fig. 11a, for each silica coverage, the decrease in reflectance was observed with increasing silica coverage and the reflectance was increased slightly more than 45%. From Fig. 11a, the *ΔpJ*_sc_ was calculated as shown in Fig. 11b. *Δ*p*J*_sc_ became the maximum value of 1.55 mA/cm^2^ at the silica coverage of 45%. Figure 12a, b, and c show SEM images of the molds prepared with the silica coverage of 16, 45, and 57%. The shape of LTSs was dome-shape, since the tet was 5 min. From the SEM images, the fraction of a flat area was decreased with increasing the silica coverage. Since a large flat area increases the fraction of non-scattering light, it leads to the high reflectance. However, the reflectance was almost same above 45%. The flat area with the size around wavelength of incident light has scattering effect such as Mie scattering. It was speculated that such a scattering became prominent around the 45%. When the flat area became smaller further, the effect of the Mie scattering became smaller, leading to the slight decrease in the reflectance. As silica coverage is increased, the aggregation of LTSs became prominent. The aggregation is caused by the solvent. It is believed that if solvents that evaporate slowly are used, the degree of dispersion of the silica particles is increased and the light scattering effect can be enhanced. The p*J*_sc_ was improved from 2.34 to 3.89 mA/cm^2^, which corresponds to the improvement by 66%. This leads to the significant contribution to the improvement of *J*_sc_ in thin crystalline silicon solar cells.

## Conclusion

Submicron-sized LTSs were fabricated by a nanoimprint method. Before nanoimprinting, master molds with submicron-sized patterns were prepared by silica colloidal lithography and reactive ion etching. By controlling silica coverage, *t*_et_ and *D*, the density, height, and size of LTSs could be controlled. Regardless of *D*, the height of LTSs has a maximum with respect to *t*_et_. As *D* increases, the *t*_et_ when the height of LTSs becomes maximum (*t*_h-max_) increases. The SEM image revealed that the LTSs were changed from a dome-shape to a cone-shape around *t*_et_ = *t*_h-max_. Around *t*_et_ = *t*_h-max_, silica particles started to disappear on the LTSs. By the absence of etching masks, the LTSs themselves were etched and the shape was changed and the height of the LTSs was decreased. The reflectance of c-Si/ITO/LTSs/Electrode structure was evaluated by spectrophotometer. The reflectance measurement showed that the LTSs reduced the reflectance in the wavelength range from 1000 to 1200 nm compared to the sample without LTSs. This indicates that the fabricated LTSs exhibit optical confinement performance. The *Δ*p*J*_sc_ was calculated from the measured reflectance. Lower aspect ratio leads to higher *Δ*p*J*_sc_. A maximum gain of 1.00 mA/cm^2^ was observed at *D* = 800 nm and *t*_et_ = 5 min, suggesting that *D* = 800 nm may be the optimal width for the near-infrared light in the range from 1000 to 1200 nm. Furthermore, The p*J*_sc_ was improved from 2.34 to 3.89 mA/cm^2^, which corresponds to the improvement by 66%. This leads to the significant contribution to the improvement of *J*_sc_ in thin crystalline silicon solar cells.

## Supplementary Information

Supplementary file1

## Data Availability

All data supporting the conclusions of this article are included within the article.

## List of abbreviations

VIPV: Vehicle-integrated photovoltaics
LTS: Light trapping structure
*D*: Diameter of silica particles
*t*_et_: Etching time
c-Si: Crystalline Si
ZnO: Zinc oxide
ITO: Indium tin oxide
RF: Radio-frequency
DI: Deionized
*J*_sc_: Short-circuit current density
*F*_c__a__p_: Capillary force
*t*_h-max_: *t*_Et_ when the height of LTSs becomes maximum
FDTD: Finite-difference time domain
Ref: Reflectance spectra of the structure of c-Si/ITO/electrode
*t*_r-min_: *t*_Et_ when the reflectance was reduced locally in the whole range
G1: Low silica coverage group
G2: High silica coverage group
p*J*_sc_: Projected *J*_sc_
*Δ*p*J*_sc_: Increment of p*J*_sc_ against the Ref sample
