# Supplementary material for: Fabrication of light trapping structures specialized for near-infrared light by nanoimprinting for the application to thin crystalline silicon solar cells
Source: Discov Nano. 2023 May 3;18(1):72. doi: 10.1186/s11671-023-03840-6 (PMC10214916; doi:10.1186/s11671-023-03840-6)
Supplement: Supplementary file 1 — Supplementary file1 [file 11671_2023_3840_MOESM1_ESM.docx]

Supporting Information

**Fabrication of light trapping structures specialized for near-infrared light by nanoimprinting for the application to thin crystalline silicon solar cells**

^*^Yuto Kimata^1^, Kazuhiro Gotoh^1^, Satoru Miyamoto^1^, Shinya Kato^2^, Yasuyoshi Kurokawa^1^, and Noritaka Usami^1^

1: Department of Materials Process Engineering, Nagoya University, Nagoya 464-8603, Japan

2: Department of Electrical and Mechanical Engineering, Nagoya Institute of Technology, Showa-ku, Nagoya, 466-8555, Japan

^*^E-mail: [kimata.yuto.w2@s.mail.nagoya-u.ac.jp](mailto:kimata.yuto.w2@s.mail.nagoya-u.ac.jp), kurokawa.yasuyoshi@material.nagoya-u.ac.jp

Figure S1. SEM images of (a) the master mold and (b) the LTSs transferred to the sample. The shape, width, and height of the LTSs correspond to those of the master mold, suggesting that nanoimprinting process was successfully conducted.

Figure S2. SEM images of deposited silica particles at the position of 0 mm, 5 mm, and 10 mm from the center of the substrate. The particle size was 800 nm and the rotation speed was fixed at 8000 rpm. From these SEM images, the silica coverage was calculated as shown in Fig. 2. The deposition should be generally uniform from the center to the edges of the substrate. The silica coverage was increased with increasing the silica concentration.

Figure S3. (a) Optical simulation model and (b) calculated photon absorption rate of silicon heterojunction solar cells implemented with LTSs. This calculation was conducted by finite difference time domain (FDTD) method. The intensity and wavelength were 0.1 W/cm^2^ and 1000 nm, respectively. The width (*W*_LTS_), height (*h*_LTS_), filling factor (*ρ*_LTS_) of LTSs were 800 nm, 100 nm, and 0.5, respectively. The Shockley-Read-Hall recombination and Auger recombination were taken in account in this simulation.

Figure S4. Reflectance spectra with and without LTSs. The *D*, silica coverage, and *t*_et_ were 800 nm, 45%, and 5 minutes, respectively. The reflectance of the sample with the LTSs in the visible light region was almost same as that without the LTSs. To improve the absorption in the visible light region, an optical confinement structure should be implemented on the front side as well.

Table S1. Calculation results of reflectance and internal quantum efficiency

| Wavelength  (nm) | Reflectance  (%) | Internal quantum efficiency  (%) |
| --- | --- | --- |
| 1000 | 32.9 | 81.1 |
